# Preparation of Graphene-Modified Acupuncture Needle and Its Application in Detecting Neurotransmitters

**DOI:** 10.1038/srep11627

**Published:** 2015-06-26

**Authors:** Lina Tang, Danxin Du, Fan Yang, Zhong Liang, Yong Ning, Hua Wang, Guo-Jun Zhang

**Affiliations:** 1 School of Laboratory Medicine, Hubei University of Chinese Medicine, 1 Huangjia Lake West Road, Wuhan 430065, China; 2 School of Acupuncture and Moxibustion, Hubei University of Chinese Medicine, 1 Huangjia Lake West Road, Wuhan 430065, China; 3 Hubei Provincial Collaborative Innovation Center of Preventive Treatment by Acupuncture and Moxibustion, 1 Huangjia Lake West Road, Wuhan 430065, China

## Abstract

We report a unique nanosensing platform by combining modern nanotechnology with traditional acupuncture needle to prepare graphene-modified acupuncture needle (G-AN), and using it for sensitive detection of neurotransmitters via electrochemistry. An electrochemical deposition method was employed to deposit Au nanoparticles (AuNPs) on the tip surface of the traditional acupuncture needle, while the other part of the needle was coated with insulation paste. Subsequently, the G-AN was obtained by cyclic voltammetry reduction of a graphene oxide solution on the surface of the AuNPs. To investigate the sensing property of the G-AN, pH dependence was measured by recording the open circuit potential in the various pH buffer solutions ranging from 2.0 to 10.0. What’s more, the G-AN was further used for detection of dopamine (DA) with a limit of detection of 0.24 μM. This novel G-AN exhibited a good sensitivity and selectivity, and could realize direct detection of DA in human serum.

Acupuncture is a technique of inserting and manipulating acupuncture needles into the body’s acupoints for the purpose of relieving pain and realizing disease therapy^1^,^2^. Acupuncture originated in China and was considered as a typical assay in traditional Chinese medicine (TCM). The first written reference to acupuncture occurred in the Huang Dei Nei Ching (The Yellow Emperor’s Classic of Internal Medicine), dating back at least 2400 years. In describing acupuncture technique, initially two terms were used: (1) jing luo, meaning “conduits” or “meridians”; and (2) xue, meaning “holes”. The meridians is a network which can regulate human body, meanwhile, the holes are windows through which human body is observed^3^,^4^,^5^,^6^. Although acupuncture holds the functions of therapeutic aims, the mechanism of the acupuncture medicine remains unclear. It is believed that a molecular event usually occurs while acupuncture needles are manipulated on the body. Therefore, it is of great significance to figure out the related molecular events in acupuncture medicine.

Acupuncture needles can be made of many materials like gold, silver, copper, stainless steel, etc. Compared to other materials, stainless steel needles are quite cheap and commonly used. Because the needle body is one of metal materials and has good conductivity, acupuncture needles can be considered as an aciform sensor. So the needle not only integrates the technology of the modern sensor and traditional acupuncture, but also measures signals with little or no damage to the human body.

Early studies of acupuncture needles as sensing needles on lactate detection have been explored^7^, in which the sensing membrane of the needle was coated with polymer by dipping it in the polymer solution. Apparently, the modification is carried out through adsorption, resulting in uncontrollable membrane thickness. More importantly, the polymer is easy to peel off from the needle body while the acupuncture needles are inserted into the body. Both rough sensing surface and unstable infrastructure make the sensing needles incapable in terms of reproducibility and stability in practical applications. So there is a demand for preparation of novel acupuncture needles with a stable and thin membrane.

Graphene^8^,^9^,^10^,^11^,^12^,^13^, consisting of a one-atom-thick planar sheet comprising and sp^2^-bonded carbon structure, has fascinated many researchers in biological applications due to its unique and excellent electronic properties, thermal, high carrier mobility as well as mechanical properties and much lower synthetic costs since its discovery. Cai and coworkers^14^ showed a good sample by using graphene to modify filed-effect transistor biosensor for label-free detection of DNA, and the detection limit as low as 100 fM was achieved. Wang *et al*.^15^ fabricated nanoelectrode ensembles via electrodeposition of Au nanoparticles (AuNPs) on graphene oxide (GO) sheet which was coated on a glass carbon electrode (GCE). Liu *et al*.^16^ used a graphene-polyaniline composite film as a basement for dopamine (DA)’s high sensitive determination. Graphene is thus a good candidate of advanced materials which could be combined with other functional materials (such as AuNPs^15^,^17^,^18^,^19^, polymers^16^,^20^,^21^ etc.) to fabricate the sensing interface for electroanalysis and enhance the sensitivity and selectivity of biosensors. In addition, electrochemical biosensors are excellent methods due to their cost-effective fabrication, high sensitivity and easy handling in applications. The currently-developed electrochemical biosensors usually employ glassy carbon electrodes and gold electrodes as basements. Those electrodes are commercially available and the modification methods of nanomaterials on the surface of the electrodes have well been developed. However, combination of acupuncture needle with nanomaterials and electrochemical detection method, allowing manipulation of the acupuncture therapy and measurement of bioactive molecules at the same time, has never been reported.

DA, one of the most important neurotransmitters, has been involved in many physiological processes in mammalian central nervous system. Abnormal DA concentration in the brain may lead to serious diseases^22^,^23^,^24^. In this paper, we prepare homogeneous and stable layers of AuNPs and graphene on the tip surface of acupuncture needles, which is the first attempt to use the electrochemical method to modify nanomaterials on the surface of acupuncture needle for fabrication of graphene-modified acupuncture needle (G-AN), as illustrated in Fig.1. We then explore the G-AN’s applications in determining pH and DA, respectively. The developed G-AN shows a potential in exploring the molecular events in acupuncture medicine.

## Results and Discussion

### Properties of the graphene-modified acupuncture needle

AuNPs are electrochemically deposited on the acupuncture needles as the method is typical and commonly-used^25^,^26^,^27^ To obtain the reduced GO, electrochemical reduction of GO (ERGO) is commonly employed in electrochemical biosensors, which is attractive due to its simple, fast, inexpensive and green nature^28^,^29^,^30^,^31^,^32^. As demonstrated in our early work^33^, the one-step electrodeposition technique is an ideal method of preparing controllable and stable graphene films directly from GO dispersions by cyclic voltammetry (CV). Therefore, this preparation method is employed in our work to fabricate the G-AN.

Figure 2 (A–C) displays scanning electron microscopy (SEM) images of acupuncture needles before and after nanomaterials modification. As seen in Fig. 2A, the bare acupuncture needle had a smooth surface with a tip diameter around 10 μm. After AuNPs were electrochemically deposited onto the tip surface of the pristine acupuncture needle, the AuNPs were observed to be distributed on the acupuncture needle (Fig. 2B). The diameters of the AuNPs were appropriately tens to hundreds nanometers, and the AuNPs did not form agglomerates within the appropriate deposition time. The AuNPs deposited onto the acupuncture needle could obtain a large surface area and good electrochemical properties, proving a great interface for the next modification. After graphene deposition, it was obviously seen that graphene covered the whole surface of the AuNPs (Fig. 2C). This SEM images demonstrate that both AuNPs and graphene have been successfully modified to the G-AN as anticipated.

CV is the convenient and effective tool to monitor the electrons transmission procedure of the modified electrode^28^. The electrochemical performances of the needles, modified with different nanomaterials, were investigated by CV in the presence of external redox probe [Fe(CN)_6_]^3−/4−^. As shown in Fig. 2D, the pristine acupuncture needle had no distinct redox peak (Fig. 2D (a)), while quasi-reversible one electron redox behavior of [Fe(CN)_6_]^3−/4−^ was observed on the modified acupuncture needle (Fig. 2D (b,c)). The G-AN showed larger current response than the AuNPs/acupuncture needle, which is ascribed to the fact that graphene could significantly enhance the conductivity of the electrode to facilitate the electron-transfer. The excellent electrochemical properties of the G-AN may be attributed to the synergistic effect of graphene and AuNPs.

To investigate the stability of the prepared G-AN, the method of electrochemical CVs was again employed. The modified needles were immersed into K_3_[Fe(CN)_6_] aqueous solution and scanned for 10 cycles. Fig. 2E displays the stability of the AuNPs/acupuncture needle (Fig. 2E (a)) and the G-AN (Fig. 2E (b)), respectively. It was seen that almost each cycle overlapped with others on the two different modified needles. The experiments indicate that the prepared G-AN is extremely stable and overcomes the disadvantage of using an adsorption strategy to coat a polymer as a sensing membrane^7^.

To further demonstrate that the AuNPs and graphene won’t peel off from the needle body, two experiments were performed. Firstly, the as-prepared G-AN was immersed in PBS solution for one week and SEM was conducted to compare the modified layer before and after the treatment. As seen in Fig. S1 (A, B), the layers of AuNPs and graphene remained after immersion of the G-AN in PBS for a relatively long period. Secondly, the stability was again investigated after the G-AN was inserted into brain tissue acquired from a newborn wistar rat. The cerebral hemispheres were acquired by a tweezer from the rat, and were immediately immersed in Hanks Balanced Salt Solutions (HBSS) solution. By using the tweezer to hold the brain tissue, the acupuncture needle was able to insert to cerebral cortex (Fig. S1(C)) and it was pulled out after 5 s. It was observed from the SEM image that the graphene layer was still on the needle body (Fig. S1(D)). These experiments suggest that the AuNPs and graphene modified acupuncture needles are stable, and the modified layers are not easy to peel off from the needle body even in *in-vivo* experiment.

### PH response of the graphene-modified acupuncture needle

The classical methods for measuring pH are usually based on the use of the well-known glass electrodes. Recently, researchers have used various nanomaterials (such as carbon nanotube/polyaniline^34^,^35^, ruthenium oxide thin-film^36^, graphene^37^) modified electrodes to measure pH. These sensors showed that they were sensitive to pH, allowing the determination of pH values ranging from pH 1.0 to 13.0 and the linear correlation between pH and open circuit potential demonstrated very good linearity. However, until now, none has been reported to use the nanomaterials-based acupuncture needle to measure pH.

The pH dependence of the bare acupuncture needle, the AuNPs/acupuncture needle and the G-AN was measured by recording the open circuit potential in various pH buffer solutions ranging from 2.0 to 10.0, respectively. Before each measurement, the needles were immersed into ultrapure water in order to ensure the same starting conditions. For bare needle, the curve showed a response, but was lack of linearity (Fig. S2 (A, B)). For the AuNPs/acupuncture needle, the response curve was disordered in pH range of 7.0–10.0, and was not linear either (Fig. S2 (C, D)). However, after graphene was modified, the response curve showed a good linearity as it is well known that carbon materials would give rise to a pH response^38^, as shown in Fig. 3A. It was observed that the signal stabilized within a few seconds and kept stable during the measured time. The potential signals decreased when the value of pH were increased, which is accordance with some others’ pH sensors^34^,^35^. The excellent performance was also showed in terms of linearity (Y = 0.0209–0.0299X, R^2^ = 0.9921) (Fig. 3B). The above-mentioned linear equation exhibited that the linear pH response of sensitivity was 29.9 mV/pH, which was not the Nernstian-like behavior. The reasons for this phenomenon may probably be attributed to the absence of ionizable carboxylic and hydroxyl groups on the surface of G-AN (the hydroxyl groups and residual carboxylic groups on the surface of reduced GO have not been activated, and can not well undergo protonation and deprotonation with pH), which is in good agreement with the results reported previously^37^. However, compared with the bare acupuncture needle and the AuNPs-modified acupuncture needle, the G-AN can be protonated and deprotonated with pH for the presence of graphene. As a result, these results indicate that the G-AN is more suitable for detection of biological signal molecule in organisms than the acupuncture needle without any modification and the acupuncture needle modified just with AuNPs.

### Determination of DA

In order to achieve high sensitivity and selectivity, it is essential to optimize experimental parameters for DA detection. The effects of pH on response currents of the G-AN at 0.1 mM DA were studied by CV method. The oxidation peak currents of the G-AN were increased in the different pH buffer solutions ranging from 5.5 to 7.0 and then decreased in the different pH buffer solution ranging from 7.0 to 8.0. Therefore, the optimal pH 7.0 was chosen for subsequent electrochemical detections (Supplementary Fig. S3), which is close to the physical environment and in good agreement with Liu’s report^16^.

π–π interaction between phenyl structure of DA and two-dimensional planar hexagonal carbon structure of graphene makes the electron transfer feasible. As illustrated in Fig. 4A, the differential pulse voltammetry (DPV) signal was negligible on the pristine acupuncture needle, revealing that there is no interaction between DA and the bare acupuncture needle (Fig. 4A (a)). In comparison, the modified acupuncture needles could enhance the electrical signal of DA solution. It was seen that the G-AN (Fig. 4A (d)) showed larger current response than the AuNPs/acupuncture needle (Fig. 4A (b)) and the ERGO/acupuncture needle (Fig.4A (c)), which is attributed to the fact that the graphene and AuNPs could significantly enhance the conductivity of the G-AN to facilitate the electron-transfer. Fig. 4B shows that DPV response curve of 0.1 mM DA could obviously be seen in the presence of 1 mM ascorbic acid (AA). Selective detection was realized in completely eliminating AA, because AA oxidation here is inactive, most likely its weak π–π interaction with graphene on the G-AN.

The quantitative analysis of DA was investigated via DPV by applying various concentrations of DA on the G-AN in pH 7.0 PBS. Fig. 4C presents DPV responses of different concentrations of DA on the G-AN, in which the DPV signal increased along with the increased DA concentrations. The peak currents assigned to the oxidation of DA showed a linear response with increasing DA concentrations in the range 1–100 μM. In the inset of Fig. 4D, the linear relationship between the peak currents and DA concentrations ranging from 1 to 10 μM was represented by Ip(μA) = −0.0293C_DA_ (μM)-0.3033 with a correlation coefficient of 0.9841. The G-AN was able to detect as low as 0.24 μM DA, which was estimated using 3σ/S (σ is standard deviation of the blank signal and S is the slope of the fit line shown in the inset of Fig. 4D).

The result of the developed G-AN detection platform is compared with that of other published works (Supplementary Table S1). Li and coworkers^39^ used a graphene-Au nanoparticles nanocomposite film to modify GCE for selective detection of DA and the detection limit achieved by this method was 1.86 μM, which is higher than that of our work. Apparently, the modification in that report was carried out through physical adsorption, resulting in uncontrollable nanocomposite film thickness and lower sensitivity. Sheng *et al*.^40^ prepared an electrochemical sensor with nitrogen doped graphene, which could detect ascorbic acid, dopamine and uric acid simultaneously. The detection limit of this sensor for DA was 0.25 μM, which is similar to that of our work. Qian *et al*.^18^ fabricated a GCE electrode using Au nanoparticles decorated polypyrrole/reduced graphene oxide hybrid sheets with a low detection limit of 18.29 pM. Zhang *et al*.^41^ modified GCE with core-shell structured Fe_3_O_4_@graphene nanospheres covered by nafion, in which the obtained detection limit was as low as 0.007 μM. In these cases, signal amplification strategies were employed so that the sensitivity was enhanced. Moreover, these methods are based on GCE, which has a large size of sensing areas compared to the tip of the acupuncture needle. Nevertheless, the conventionally electrochemical methods can hardly be applied to *in-vivo* test, while the nanomaterial-based acupuncture needle will potentially measure bioactive molecules *in-vivo*.

### Practical application

The practicability of the sensing needle in real sample was tested, which shows satisfactory results. As shown in Fig. 5, a negligible signal variation was achieved after the G-AN was interacted either in PBS buffer or human serum sample without DA, and the weaker signals were obtained in serum despite its extremely complex and multicomponent nature. Nevertheless, the signals obviously increased when the DA was added to the PBS buffer or serum solution. The results demonstrate that the G-AN may have practical applications in real clinical samples.

## Conclusion

In summary, we have developed a novel graphene-modified acupuncture needle biosensor by using layer-to-layer electrochemical deposition of AuNPs and graphene on the tip surface of acupuncture needle, respectively. The prepared G-AN showed a superior stability due to the nanomaterial-based infrastructures. The G-AN’s pH dependence was measured by recording the open circuit potential, and its excellent performance was achieved. What’s more, the G-AN was further used for detection of DA with a limit of detection of 0.24 μM, and the G-AN also exhibited a good selectivity. The G-AN was also successfully used to test DA in human serum samples. Compared to the previously prepared acupuncture sensing needles, the developed G-AN has the following merits: 1) Stable sensing membrane, AuNPs and graphene are conjugated to the surface of acupuncture needle via electrochemical approach, hence the sensing membrane is stable even after the graphene-modified needle is immersed in solution environment or inserted into tissue. Moreover, the G-AN presents a stable electrochemical signal after 10 cycles of scans; 2) High selectivity, the G-AN is capable of discriminating DA and AA; 3) High sensitivity, the G-AN is able to detect DA as low as 0.24 μM. Thus, this study offers a new and reliable method for preparation of G-AN, which can be used for detecting DA, providing a significant technical support in the study of acupuncture.

The ultimate goal of the developed G-AN is that it will be able to be used for *in-vivo* measurements of the molecular events while the acupuncture needles are manipulated on the human body. To do so, it will, however, involve a number of issues like: 1) Medical ethics; 2) Evaluation of toxicity on the nanomaterials used for coating of the G-AN; 3) Complexity of the *in-vivo* measurement; 4) Compatibility of TCM and modern medicine. Nonetheless, this work opens up a new field of combining nanotechnology with traditional acupuncture medicine, and provides a potential tool in understanding complex nature of patient response to acupuncture.

## Methods

### Reagents and materials

Acupuncture needles were purchased from Suzhou medical supplies factory Co. Ltd. (Suzhou, China). Nature graphite (99.95%, 325 mesh), purchased from Alfa Aesar Co. Ltd. (Tianjin, China), was used for synthesis of graphene oxide. DA and AA were obtained from aladdin (Shanghai, china). Epoxy resin, purchased from Nanjing (Nanjing, China), was used as insulator. The other reagents were purchased from Sinopharm Chemical Reagent Co. Ltd. (Shanghai, China). Serum sample was obtained from Wuhan Third Hospital (Wuhan, China). All reagents were of analytical grade and were used as received without further purification. Ultrapure water prepared by Milli-Q System (Millipore, Billerica, MA, USA) was used throughout the experiment.

### Apparatus

CV and DPV were performed on a CHI 660D electrochemical workstation (CH Instruments, Chenhua Co. Ltd., Shanghai China). A conventional three-electrode system, which was composed of a bare or modified acupuncture needle as a working electrode, a Ag/AgCl electrode as a reference electrode and a platinum wire as an auxiliary electrode, was employed throughout the experiment. Scanning electron microscopy images were obtained from a Zeiss Ultra Plus FE-SEM (Zeiss, Germany). All measurements were carried out at a room temperature.

### Preparation of the graphene-modified acupuncture needle

**Figure 1:**
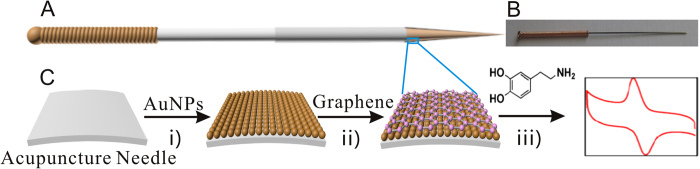
The fabrication process for the preparation of the G-AN and the detection of DA by the G-AN. (**A**) The schematic plot of a G-AN; (**B**) The optical image of an acupuncture needle; (**C**) (i) Modification of AuNPs on the tip surface of the acupuncture needle; (ii) Deposition of graphene via electrochemistry method; (iii) Measurement of DA using the G-AN. All parts of this figure were drawn by FY. The optical image of the acupuncture needle was taken by LT.

**Figure 2:**
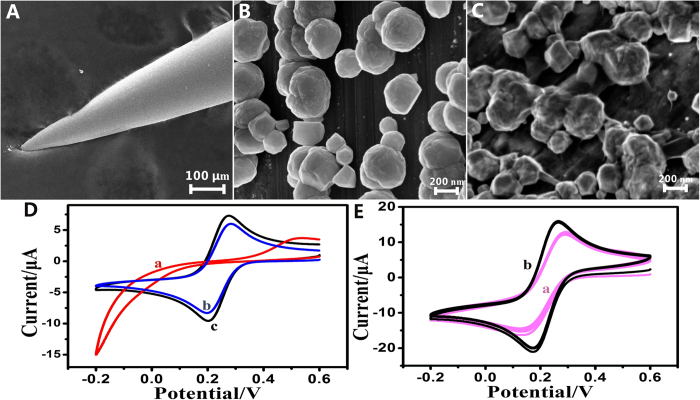
SEM images of the (**A**) bare acupuncture needle, (**B**) AuNPs/acupuncture needle, and (**C**) G-AN. (**D**) Typical CV obtained at the different nanomaterial-modified acupuncture needle ((**a**) bare acupuncture needle, (**b**) AuNPs/acupuncture needle, (**c**) G-AN, in aqueous solution consisting of 5 mmol/L of K_3_[Fe(CN)_6_] and 0.1 mol/L of KCl. (**E**) CVs for acupuncture needle with 10 cycles ((**a**) modified by AuNPs, (**b**) modified by ERGO/AuNPs). The voltage range: −0.2 to 0.6 V; scan rate: 100 mV/s.

**Figure 3:**
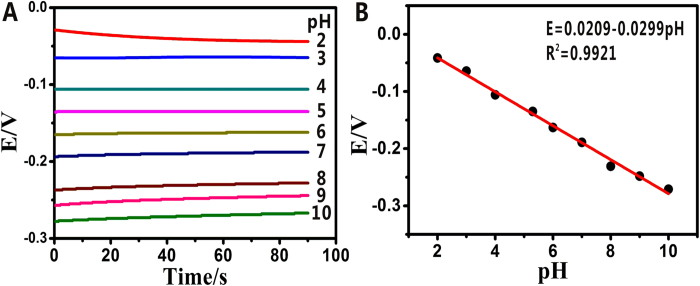
(**A**) Time dependence of the open circuit potential of the G-AN in PBS solutions at different pH values, (**B**) Plots of the potential versus various pH. Y = 0.0209–0.0299X, R^2^ = 0.9921.

**Figure 4:**
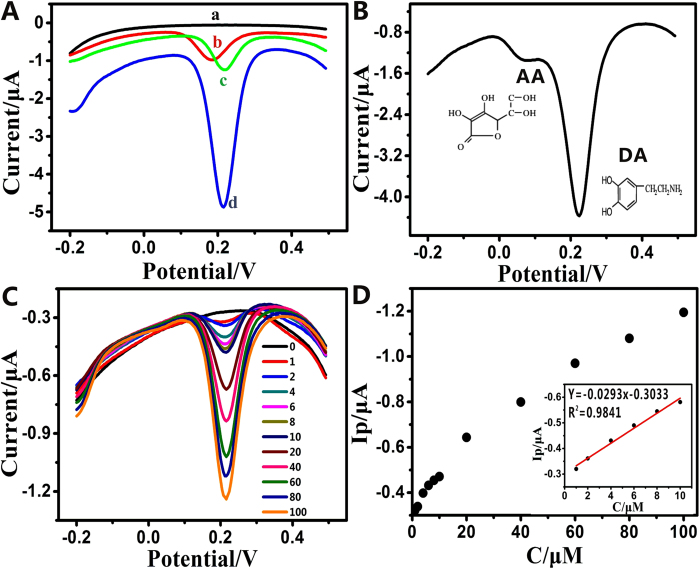
(**A**) DPVs of different acupuncture needles in the solution of 0.1 mM DA ((**a**) bare acupuncture needle, (**b**) AuNPs/acupuncture needle, (**c**) ERGO/acupuncture needle and (**d**) G-AN). (B) DPVs of the G-AN in the solution of 0.1 mM DA and 1 mM AA. Scan rate, 50 mV/s. (**C**) The DPVs versus increasing DA concentrations of 0, 1, 2, 4, 6, 8, 10, 20, 40, 60, 80 and 100 μM (from top to bottom). (**D**) In the inset, the calibration curve of DPVs versus DA concentrations (1 to 10 μM) was represented by Ip(μA) = −0.0293C _DA_ (μM)-0.3033, R^2^ = 0.9841.

**Figure 5:**
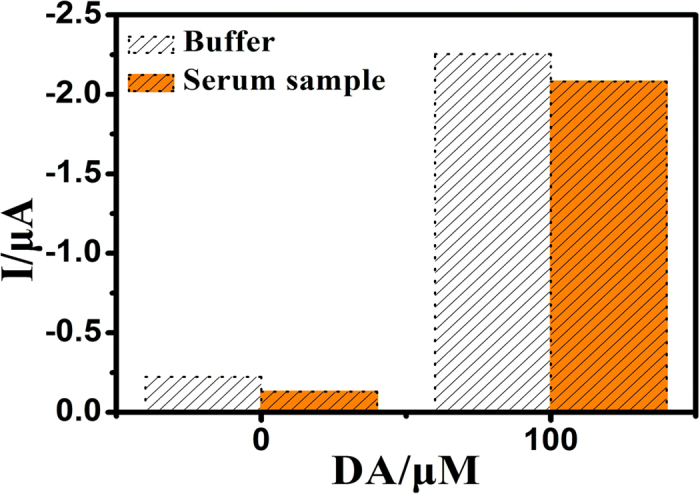
Current responses for the G-AN in the detection of DA (100 μM) in buffer and human serum.

The process for the preparation of the G-AN is showed in detail in Fig. 1. Before surface modification, acupuncture needles were rinsed and sequentially sonicated in ultrapure water, and anhydrous ethanol, respectively, for 10 min and dried in N_2_. AuNPs were deposited to the acupuncture needles by employing an electrochemical approach. Prior to electrochemical deposition, gold citrate salt solution as plating electrolytes were heated to 50 °C and this temperature condition was maintained during the electroplating experiments. AuNPs were electrochemically deposited onto the acupuncture needle to achieve AuNPs/acupuncture needle. Then the needle was rinsed, dried in N_2_. An insulated epoxy resin layer was then coated on the needle body to block the signal degradation outside the sensing membrane. After that, AuNPs/acupuncture needle was dried with air for the next modification.

The graphene modification on the AuNPs-assembled acupuncture needle was achieved by electrochemical methods according to the literatures^33^,^42^. The as-prepared GO solution was deoxygenated by bubbling pure nitrogen for at least 5 min and maintained under nitrogen atmosphere during the experiments. The CV reduction was performed in the GO dispersion on the electrochemical station. The CV was conducted from 0.5 to −1.5 V with a scan rate 50 mV/s. After deposition, the prepared acupuncture needle was rinsed with water and then dried with N_2_ to get the G-AN.

### Response of pH

The G-AN’s pH dependence was measured by recording the open circuit potentials in various PBS buffer solutions, ranging from pH 2.0 to 10.0. The pH values of all buffer solutions were controlled with a commercial pH sensor. The open circuit potential versus a saturated calomel electrode (SCE) was measured while the G-AN was dipped into the buffer solution. Before each measurement, the sensor was immersed in ultrapure water in order to ensure the same starting conditions.

### Detection of DA

To carry out the electrochemical detection, both CV and DPV were employed in a three-electrode cell in 0.1 M PBS. CVs were performed in the range of −0.2 ~ 0.6 V at a scan rate of 100 mV/s. The electrochemical parameters for DPVs were: −0.2 ~ 0.5 V, step potential, 0.004 V; modulation amplitude, 40 mV; pulse width, 0.05 s; pulse period, 0.2 s. The practical applications in serum sample were conducted by the standard addition method. PBS and serum (diluted 10 times with 0.1 M PBS) were spiked with 0.1 mM DA as experimental group. PBS and diluted serum without DA were used as control.

## Additional Information

**How to cite this article**: Tang, L. *et al*. Preparation of Graphene-Modified Acupuncture Needle and Its Application in Detecting Neurotransmitters. *Sci. Rep*. **5**, 11627; doi: 10.1038/srep11627 (2015).

## Supplementary Material

Supplementary Information
