# Supplementary material for: Preparation of Graphene-Modified Acupuncture Needle and Its Application in Detecting Neurotransmitters
Source: Sci Rep. 2015 Jun 26;5:11627. doi: 10.1038/srep11627 (PMC4481527; doi:10.1038/srep11627)
Supplement: Supplementary Information [file srep11627-s1.doc]

**Supplementary Information**

**Preparation of Graphene-Modified Acupuncture Needle and Its Application in Detecting Neurotransmitters**

Lina Tang^1^, Danxin Du^1^, Fan Yang^1^, Zhong Liang^2^, Yong Ning^1^, Hua Wang^2, 3^, Guo-Jun Zhang^1, 3^, ^*^

*^1^School of Laboratory Medicine, Hubei University of Chinese Medicine,*

*1 Huangjia Lake West Road, Wuhan 430065, China*

*^2^School of Acupuncture and Moxibustion, Hubei University of Chinese Medicine,*

*1 Huangjia Lake West Road, Wuhan 430065, China*

*^3^Hubei Provincial Collaborative Innovation Center of Preventive Treatment by Acupuncture and Moxibustion, 1 Huangjia Lake West Road, Wuhan 430065, China*

**Corresponding author: Tel: +86-27-68890259, Fax: +86-27-68890259*

*Email:* [zhanggj@hbtcm.edu.cn](mailto:zhanggj@hbtcm.edu.cn)


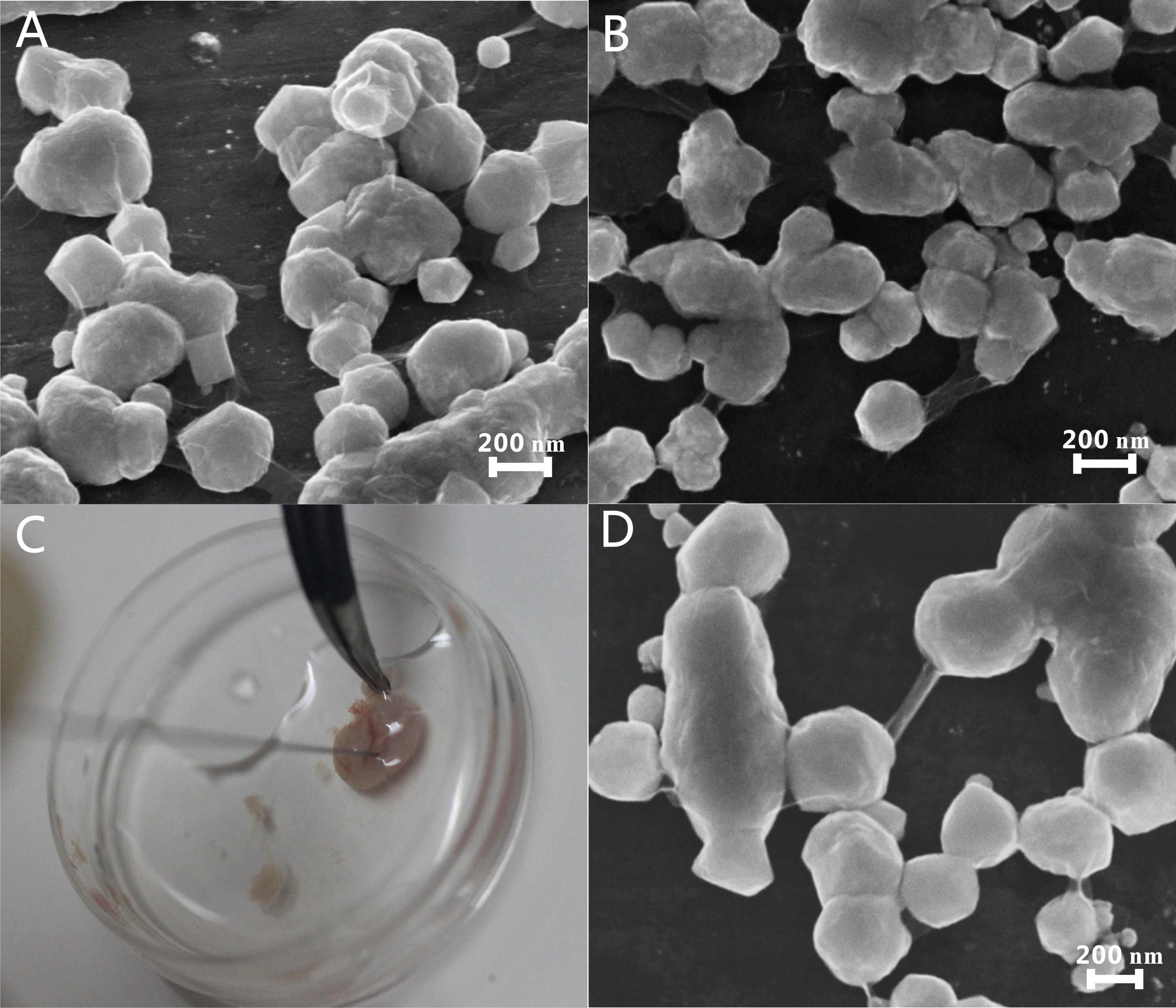


**Figure S1**. The SEM images of G-AN before (A) and after (B) immersed into PBS solution for one week. (C) The optical image of G-AN inserted into the brain tissue of a wistar rat. (D) The SEM image of G-AN taken after it was inserted into the brain tissue.


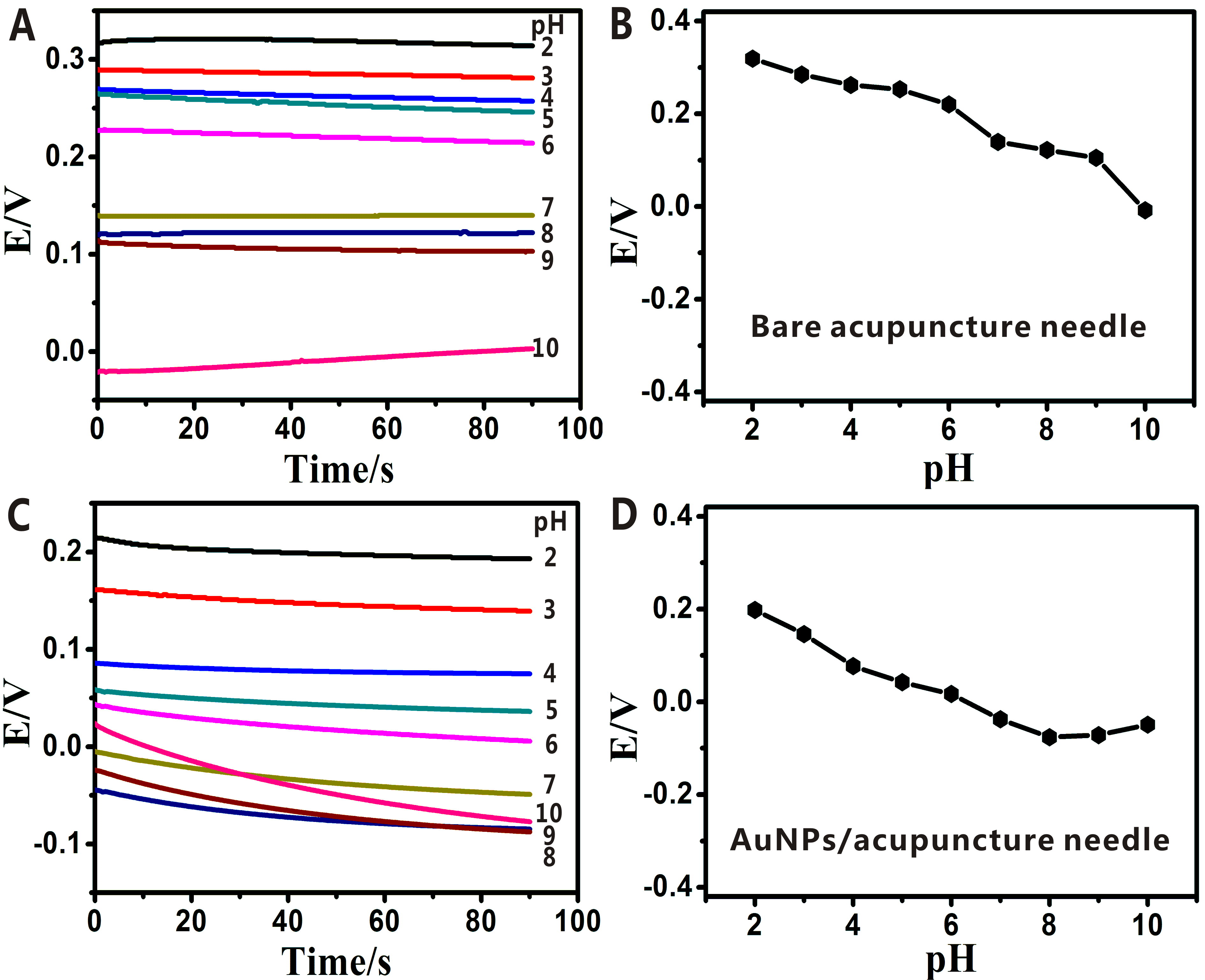


**Figure S2**. (A) Time dependence of the open circuit potential of bare acupunture needle, (B) Plots of the potential versus various pH values of bare acupunture needle, (C) Time dependence of the open circuit potential of AuNPs/acupunture needle, (D) Plots of the potential versus various pH values of AuNPs/acupunture needle.

**
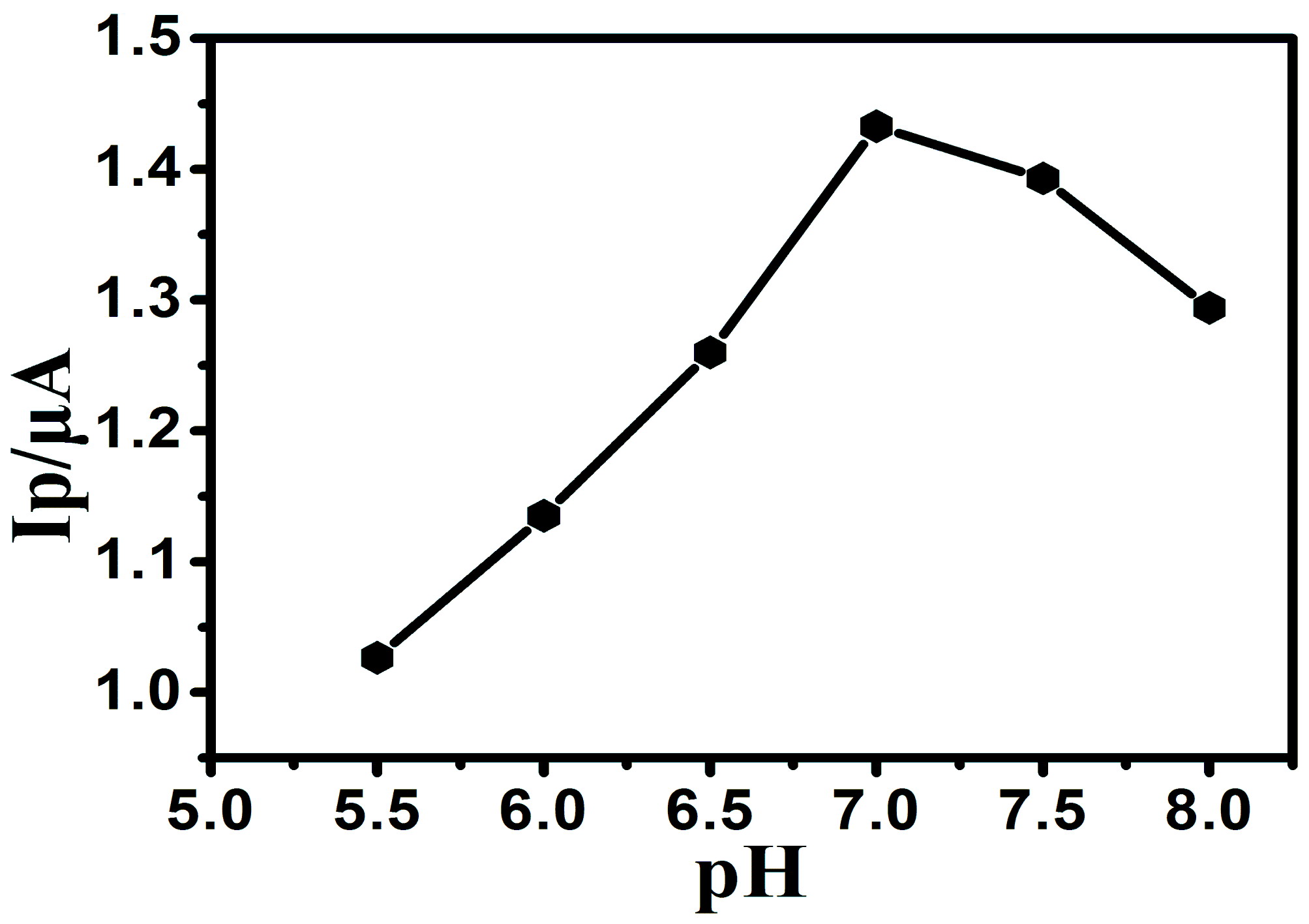
**

**Figure S3.** The relationship of oxidation peak current versus pH (pH values 5.5, 6.0, 6.5, 7.0, 7.5 and 8.0, respectively).

**Table 1.** Comparison of the proposed G-AN with other electrochemistry methods in detecting DA

| Basement | Modified materials | Detection limit(μM) | Linear range(μM) | Method | References |
| --- | --- | --- | --- | --- | --- |
| Glassy carbon | AuNPs-PPY-rGO ^a^ | 18.29×10^-6^ | 0. 0001-5 | DPV | [18] |
| Glassy carbon | GH-AuNPs ^b^ | 1.86 | 5-1000 | DPV | [39] |
| Glassy carbon | NG^c^ | 0.25 | 0.5-170 | DPV | [40] |
| Glassy carbon | Fe_3_O_4_@graphene nanospheres ^d^ | 0.007 | 0.02-130 | DPV | [41] |
| Glassy carbon | HAu-G^e^ | 0.05 | 0.08-600 | Amperometry | [S1] |
| Glassy carbon | FRGO/AuNPs ^f^ | 0.02 | 1-60 | DPV | [S2] |
| Carbon fiber electrode | RGO | 0.77 | 1.5-224.8 | DPV | [S3] |
| Glassy carbon | rGO/TiO_2_ | 6 | 2-60 | DPV | [S4] |
| Acupuncture needle | ERGO/AuNPs | 0.24 | 1-100 | DPV | This work |

^a^ Au nanoparticles decorated polypyrrole/reduced graphene oxide hybrid sheets

^b^ Graphene–Au nanoparticles nanocomposite film

^c^ Nitrogen doped graphene

^d^ core–shell structured Fe_3_O_4_@graphene nanospheres covered by nafion

^e^ Highly dispersed hollow gold-graphene nanocomposites

^f^ Functionalized reduced graphene oxide/gold nanoparticles

**References**

1. Zhu, W. C., Chen, T., Ma, X. M. & Chen, S. H. Highly sensitive and selective detection of dopamine based on hollow gold nanoparticles-graphene nanocomposite modified electrode. *Colloid. Surface. B* **111**, 321-326 (2013).
2. Liu, S. *et al*. Layer-by-layer assembled multilayer films of reduced graphene oxide/gold nanoparticles for the electrochemical detection of dopamine. *J. Electroanal. Chem.* **672**, 40-44 (2012).
3. Yang, B. B. *et al*. Direct electrodeposition of reduced graphene oxide on carbon fiber electrode for simultaneous determination of ascorbic acid, dopamine and uric acid. *Colloids and Surfaces* *A: Physicochem. Eng.* *Aspects* **456**, 146-152 (2014).
4. How, G. T., Pandikumar, A., Ming, H. N. & Ngee, L. H. Highly exposed {001} facets of titanium dioxide modified with reduced graphene oxide for dopamine sensing. *Sci. Rep.* **5044**,1-8 (2014)
